# Avian Influenza A(H9N2) Virus in Poultry Worker, Pakistan, 2015

**DOI:** 10.3201/eid2501.180618

**Published:** 2019-01

**Authors:** Muzaffar Ali, Tahir Yaqub, Nadia Mukhtar, Muhammad Imran, Aamir Ghafoor, Muhammad Furqan Shahid, Muhammad Naeem, Munir Iqbal, Gavin J.D. Smith, Yvonne C.F. Su

**Affiliations:** University of Veterinary and Animal Sciences, Lahore, Pakistan (M. Ali, T. Yaqub, M. Imran, A. Ghafoor, M.F. Shahid);; Health Care Department, Government of Punjab, Lahore (N. Mukhtar);; Bahauddin Zakariya University, Multan, Pakistan (M. Naeem);; The Pirbright Institute, Compton Laboratory, Newbury, UK (M. Iqbal);; Duke University, Durham, North Carolina, USA (G.J.D. Smith);; Duke-National University Singapore Medical School, Singapore (G.J.D. Smith, Y.C.F. Su)

**Keywords:** Surveillance, avian influenza, influenza A(H9N2), zoonoses, evolution, viruses, influenza, Pakistan, poultry workers

## Abstract

Avian influenza A(H9N2) virus isolated from a poultry worker in Pakistan in 2015 was closely related to viruses detected in poultry farms. Observed mutations in the hemagglutinin related to receptor-binding affinity and antigenicity could affect cross-reactivity with prepandemic H9N2 vaccine strains.

Influenza A(H9N2) virus circulates in domestic poultry, and outbreaks have been recorded since the early 1990s in China (*1*). In Pakistan, H9N2 virus was first detected in a poultry outbreak in 1998; subsequent outbreaks have led to increased genetic diversifications of distinct viral lineages descended from H9N2 G1 lineage viruses (*2*). Serologic studies of H9 virus among persons of different occupations in Pakistan who had direct exposure to poultry (e.g., poultry workers, vaccinators, veterinarians) have shown high rates of seropositivity (30%–85%) (*3*–*5*). Although no human infection with H9N2 virus has been reported from Pakistan, sporadic clinical cases of H9N2 virus infection in humans have been reported in China (*6*), Hong Kong (*7*), and Bangladesh (*8*). We report the isolation of H9N2 virus from a poultry worker during avian influenza virus surveillance in Pakistan.

## The Study

During January 2015–June 2016, avian influenza virus (AIV) surveillance was conducted in poultry farms throughout 19 districts of Punjab Province, Pakistan (*9*). In addition, after obtaining written informed consent, we collected 117 nasal swab specimens from male poultry workers 25–35 years of age. The Institutional Ethical Review Board at the Institute of Public Health (Lahore, Pakistan) reviewed and approved the study protocol.

Sterile swabs were used to take nasal swab samples from humans; the swabs were placed in sterile tubes containing 2 mL of viral transport media. To prevent cross-contamination between samples, human and chicken samples were collected in different zip-sealable plastic bags and transported on ice to the laboratory. Human and chicken samples were also processed and cultured separately on different dates. Individual human samples were inoculated in 9-day-old embryonated chicken eggs and amnio-allantoic fluid (AAF) harvested after 48 h incubation. Harvested AAF was first tested by hemagglutination assay, and positive AAF screened for H5, H7, H9, and Newcastle disease virus (NDV) using hemagglutination inhibition (HI) assay as previously described (*9*).

We detected 1 H9-positive sample collected from a poultry worker in Narowal District, Punjab, where only 1 (1.1%) of 88 chicken samples was H9N2-positive (*9*), and the flock from the same farm was H9 negative. The 36-year-old worker did not display major signs of influenza-like illness, which is typical of the mild to no symptoms shown in H9N2 virus infection (*10*). No human samples were positive for H5 or H7 virus. The virus isolate was confirmed as H9N2 by reverse transcription PCR, and the hemagglutinin (HA) and neuraminidase (NA) genes were sequenced as described (*9*). We sequenced the HA and NA genes of 8 additional viruses isolated from chickens in different districts of Punjab (Table 1).

**Table 1:** H9-HA and N2-NA influenza virus sequences isolated from poultry and a human, Punjab, Pakistan, 2015–2016

Virus isolate	Collection date	Host	District	GenBank accession no.	
				HA	NA
A/chicken/Pakistan/12CF/2015	2015 May 19	Commercial chicken	Lahore	MH930826	MH930508
A/chicken/Pakistan/540CF/2015	2015 Jun 8	Commercial chicken	Gujranwala	MH930827	MH930509
A/chicken/Pakistan/740CF/2015	2015 May 29	Commercial chicken	Layyah	MH930828	MH930510
A/chicken/Pakistan/870CF/2015	2015 Jul 11	Commercial chicken	Sharqpur	MH930829	MH930511
A/chicken/Pakistan/1108CF/2016	2016 Apr 30	Commercial chicken	Rawalpindi	MH930830	MH930512
A/chicken/Pakistan/401BYP/2015	2015 Jun 30	Backyard chicken	Narowal	MH930831	MH930513
A/chicken/Pakistan/654BYP/2015	2015 Dec 12	Backyard chicken	Rawalpindi	MH930832	MH930514
A/chicken/Pakistan/800BYP/2016	2016 Jun 26	Backyard chicken	Sharqur	MH930833	MH930515
A/Pakistan/486/2015	2015 Nov 19	Human	Narowal	MH930834	MF280171

*HA, hemagglutinin; NA, neuraminidase.

We analyzed 2,751 H9NX and 8,059 HXN2 avian and human virus sequences (collected during 1963–2017) obtained from GenBank and the GISAID database (https://platform.gisaid.org) and reconstructed large H9-HA and N2-NA phylogenies using maximum-likelihood (ML) analysis. We then subsampled the datasets to a final dataset of 145 sequences for each H9-HA and N2-NA. Reconstruction of temporal phylogenies was performed as previously described (*11*). H9-HA (Figure; Appendix Figure 1) and N2-NA (Appendix Figure 2) phylogenies show that, within the G1 lineage, the human H9N2 isolate (A/Pakistan/486/2015) formed a strongly supported monophyletic group with chicken H9N2 viruses collected in Pakistan from 2015 to 2016. The HA and NA genes of A/Pakistan/486/2015 (H9N2) showed close genetic resemblance (99.8%–99.9% nt identity) with 2015–2016 Pakistan chicken viruses, most likely indicating direct cross-species transmission from poultry to human. These 2015–2016 Pakistan viruses are further grouped within sublineage B2 (*2*), which also includes viruses isolated during 2008–2014 from Afghanistan, Iran, and Pakistan. The mean times to the most recent common ancestor (tMRCAs) of the HA and NA genes for the 2015–2016 Pakistan strains were both estimated as late 2010 (Table 2), and sublineage B2 mean tMRCAs were estimated as 2005–2006. Together, these results indicate the circulation of unsampled virus diversity in poultry during the past decade, hindering our ability to investigate virus transmission and evolution of H9N2 virus in Pakistan and highlighting the need for systematic surveillance.

**Figure:**
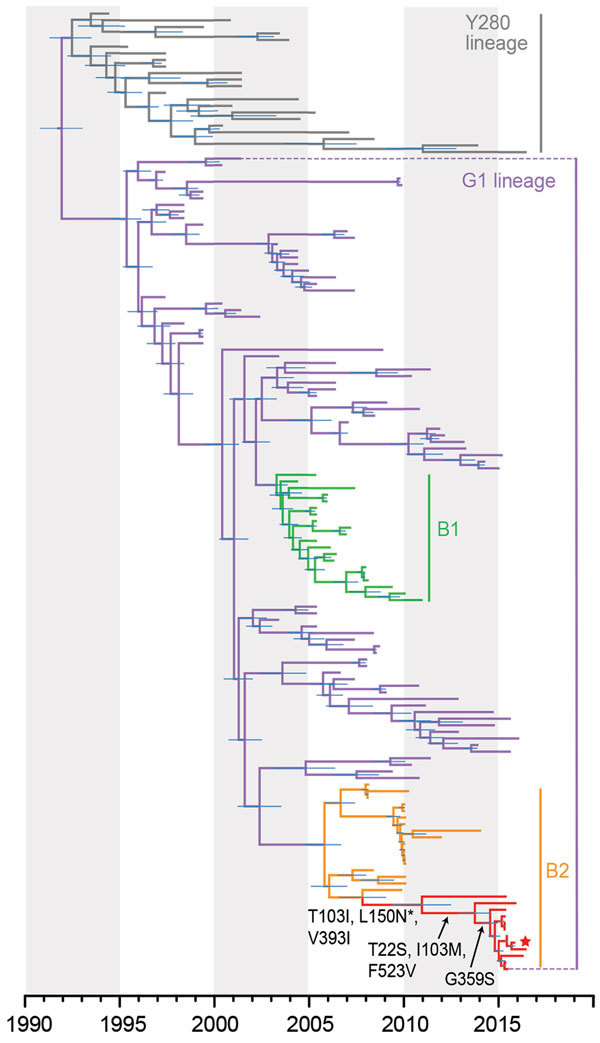
Evolutionary relationships of the influenza A virus H9-HA gene isolated from avian and human hosts, Pakistan, 1998–2016. The phylogeny was generated using the uncorrelated lognormal relaxed molecular clock, the SRD06 codon position model, HKY85 plus gamma substitution model, and a Gaussian Markov random field (GMRF) Bayesian skyride in BEAST version 1.8.4. Two independent runs of 100 million Markov chain Monte Carlo generations were performed. Horizontal node bars represent the 95% highest posterior density intervals. Red branches indicate new sequences generated from this study, and the new human isolate is marked by a red star. Black arrows indicate the amino acid mutations (H9 numbering) for the 2015–2016 Pakistan lineage, and asterisk indicates site under positive selection.

**Table 2:** Estimated tMRCA of influenza A(H9N2) virus sublineages, Pakistan*

Gene and lineage	tMRCA (date)		
Mean	Upper 95% HPD	Lower 95% HPD
H9-HA			
Sublineage B1	2003.27 (2003 Apr 10)	2003.86 (2003 Nov 11)	2002.62 (2002 Aug 16)
Sublineage B2	2005.81 (2005 Oct 24)	2006.70 (2006 Sep 14)	2004.74 (2004 Sep 29)
2015–2016 (Pakistan)	2010.95 (2010 Dec 14)	2012.49 (2012 Jun 29)	2009.32 (2009 Apr 28)
N2-NA			
Sublineage B2	2006.84 (2006 Nov 4)	2007.89 (2007 Nov 22)	2005.74 (2005 Sep 29)
2015–2016 (Pakistan)	2010.71 (2010 Sep 18)	2012.23 (2012 Mar 26)	2009.29 (2009 Apr 17)

*HA, hemagglutinin; HPD, highest posterior density; NA, neuraminidase; tMRCA, time to the most recent common ancestor.

All 2015–2016 Pakistan lineage viruses possessed a leucine (L) residue at position 226 (H3 numbering) in the HA receptor-binding site (RBS), which is associated with greater affinity for α-2,6 binding. However, HA residue 228 (H3 numbering) in the RBS was glutamine (G), known for avian-like receptor specificity. All 2015–2016 Pakistan lineage viruses possessed a low pathogenic motif (KSSR/G) in the HA cleavage site. To further characterize the recent evolution of H9N2 viruses in Pakistan, we mapped the HA and NA amino acid substitutions onto the phylogenetic trees and used Mixed Effects Model of Evolution (MEME) analysis to detect amino acids under positive selection, as previously described (*11*). Three amino acid substitutions were present at the ancestral node of the HA of the 2015–2016 Pakistan lineage, T103I, L150N, and V393I (mature H9 protein numbering), although only residue 150 (H3 numbering 160) was under positive selection. One additional substitution, T186A (H3 numbering 196), was present in the human isolate A/Pakistan/486/2015 (H9N2). Both of these mutations are situated in the antigenic region of the H9-HA globular head and correspond to H3N2 epitope B (*12*). The biologic function of mutations at the remaining residues is not known. In addition, the HA phylogeny also indicated that 2015–2016 Pakistan viruses had diverged from lineages containing the 3 H9N2 G1 lineage candidate vaccine viruses (A/Hong Kong/1073/99, A/Hong Kong/33982/2009, and A/Bangladesh/994/2011) (Appendix Figure 1). These vaccine candidates may therefore not provide efficient protection against the avian 2015–2016 Pakistan lineage, although this assumption must be confirmed by antigenic assays.

No deletion in the NA gene, associated with aquatic to terrestrial host adaptation and increased replication in ferrets (*13*), was observed in the 2015–2016 Pakistan lineage viruses. Numerous substitutions were observed in the 2015–2016 Pakistan lineage viruses (Appendix Figure 2). Of these, the Q39R, K47E, and I62T mutations are functionally key residues in the NA stalk that may be associated with host adaptation and virus virulence (*13*). The mutations at 372 and 401 residues may be responsible for hemadsorption activity of the NA (*14*). The functional importance of the remaining mutations is unknown, including V263I that was under positive selection.

## Conclusions

Our detection and isolation of H9N2 virus from a poultry worker in Pakistan highlights the potential for cross-species transmission of H9 viruses in the country. The World Health Organization considers avian H9N2 viruses a consistent pandemic threat because they are widespread in poultry and cause sporadic infection in humans. H9N2 viruses have been central to the generation of other viruses of pandemic concern and have contributed the internal genes to both H5 and H7 viruses in China (*15*). Although the subtype is relatively well studied in China, investigation in other countries is generally limited in scope. Within Pakistan, H9N2 viruses in chickens have circulated endemically for at least a decade, yet systematic surveillance is lacking.

Our results show continued diversification of H9N2 viruses in Pakistan; viruses isolated during 2015–2016 formed a distinct clade to earlier viruses from Afghanistan, Iran, and Pakistan isolated during 2008–2014. Dating analysis further estimated the tMRCA of the 2015–2016 Pakistan viruses as late 2010, indicating at least 5 years of unsampled virus diversity that circulated in poultry. We also observed mutations in HA related to changes in receptor-binding affinity and antigenicity that could affect cross-reactivity with the World Health Organization–recommended prepandemic H9N2 vaccine strains. None of the 3 G1 candidate vaccine viruses are closely related to strains from Pakistan.

Phylogenetic relationships indicate H9N2 virus transmission across South Asia and the Middle East, where the persistence and circulation of AIV are poorly understood. Increased surveillance in wild bird populations, poultry farms and markets, and occupationally exposed workers is needed in these regions to identify the emergence of antigenic variants and to maintain up-to-date H9 vaccine candidates.

Appendix Evolutionary relationships of the influenza virus H9-HA and N2-NA genes isolated from avian and human hosts, Pakistan, 1998–2016.
