# Supplementary material for: Avian Influenza A(H9N2) Virus in Poultry Worker, Pakistan, 2015
Source: Emerg Infect Dis. 2019 Jan;25(1):136–9. doi: 10.3201/eid2501.180618 (PMC6302575; doi:10.3201/eid2501.180618)
Supplement: Appendix — Evolutionary relationships of the influenza virus H9-HA and N2-NA genes isolated from avian and human hosts, Pakistan, 1998–2016. [file 18-0618-Techapp-s1.pdf]

Article DOI: https://doi.org/10.3201/eid2501.180618

Avian Influenza A(H9N2) Virus in Poultry Worker, Pakistan, 2015

Technical Appendix

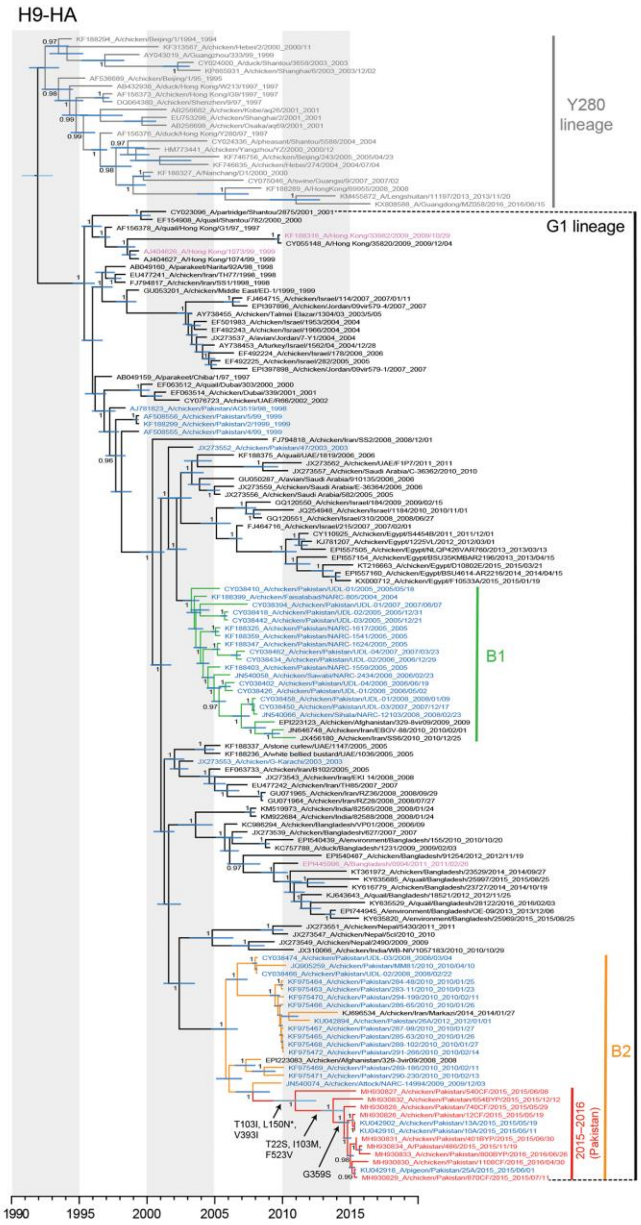

Technical Appendix Figure 1. Evolutionary relationships of the influenza A virus H9-HA gene isolated from avian and human hosts, Pakistan, 1998–2016. The phylogeny was generated using the uncorrelated

lognormal relaxed molecular clock, the SRD06 codon position model, HKY85+Γ substitution model and a Gaussian Markov random field (GMRF) Bayesian skyride in BEAST v1.8.4. Two independent runs of 100 million Markov chain Monte Carlo (MCMC) generations were performed. Horizontal node bars represent the 95% HPD intervals and posterior probability values >0.95 were indicated at nodes. Virus isolates in red indicate new sequences generated from this study, blue isolates indicate previously described Pakistan viruses; pink isolates represent vaccine strains used for H9 viruses (http://www.who.int/influenza/vaccines/virus/candidates_reagents/a_h9n2/en/). Black arrows indicate the amino acid mutations (H9 numbering) for the 2015–2016 Pakistan lineage, and asterisk indicates site under positive selection.

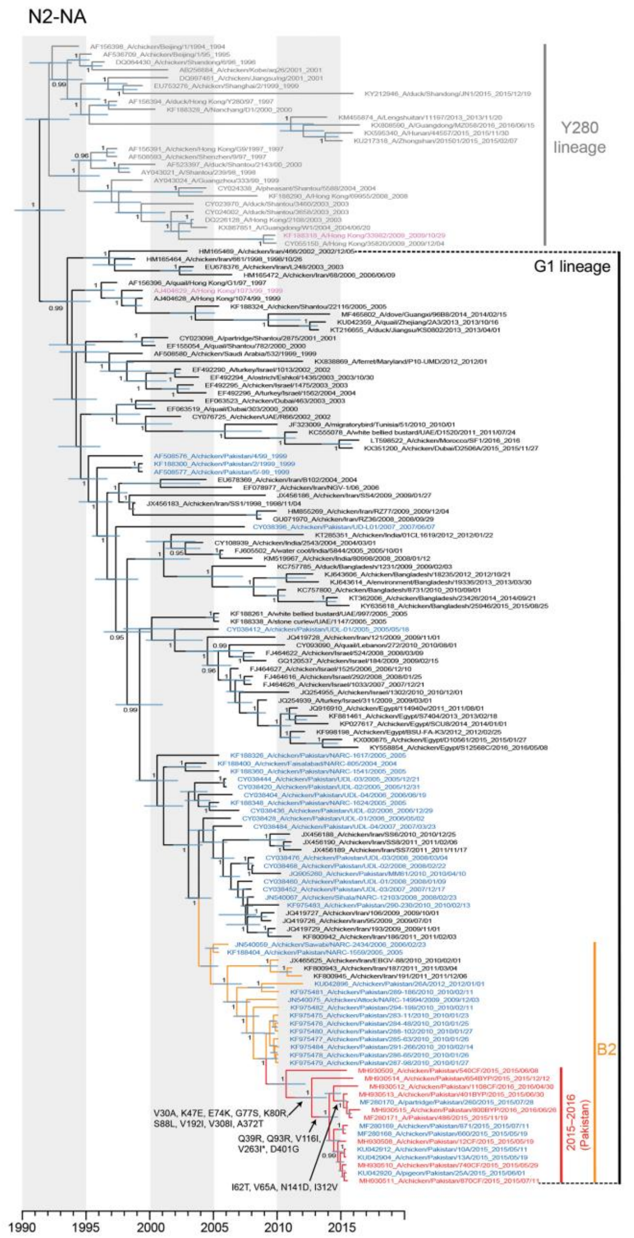

Technical Appendix Figure 2. Evolutionary relationships of the influenza A virus N2-NA gene isolated from avian and human hosts, Pakistan, 1998–2016. The phylogeny was generated using the uncorrelated lognormal relaxed molecular clock, the SRD06 codon position model, HKY85+Γ substitution model and a Gaussian Markov random field (GMRF) Bayesian skyride in BEAST v1.8.4. Two independent runs of 100 million Markov chain Monte Carlo (MCMC) generations were performed. Horizontal node bars represent the 95% HPD intervals and posterior probability values >0.95 were indicated at nodes. Virus isolates in red indicate new sequences generated from this study, blue isolates indicate previously described Pakistan viruses, and pink isolates represent vaccine strains used for H9 viruses. Black arrows indicate the amino acid mutations (H9 numbering) for the 2015–2016 Pakistan lineage and asterisk indicates site under positive selection.
